# Resistance of polio to its eradication in Pakistan

**DOI:** 10.1186/1743-422X-8-457

**Published:** 2011-10-02

**Authors:** Masaud Shah, Muhammad Kazim Khan, Saleha Shakeel, Faiza Mahmood, Zunaira Sher, Muhammad Bilal Sarwar, Aleena Sumrin

**Affiliations:** 1 National Center of Excellence in Molecular Biology, University of the Punjab, Lahore, Pakistan

**Keywords:** Polio, immunization, vaccines, EPI, Pakistan

## Abstract

**Background:**

This study is based on EPI (Expanded Program on Immunization) immunization surveys and surveillance of polio, its challenges in immunization and the way forward to overcome these challenges.

**Methods:**

Several Government documents, survey reports and unpublished program documents were studied and online search was made to find information on EPI Pakistan. SPSS 16 and Microsoft Excel 2007 were used for the statistical analysis.

**Results:**

Immunization against polio is higher in urban areas as compared to rural areas. Marked variation in vaccination has been observed in different provinces of Pakistan in the last decade. Secondly 10-20% of the children who have received their first dose of trivalent polio vaccine were deprived of their 2^nd ^and 3^rd ^dose because of poor performance of EPI and Lack of information about immunization.

**Conclusion:**

In spite of numerous successes, such as the addition of new vaccines and raising immunization to over 100% in some areas, EPI is still struggling to reach its polio eradication goals. Inadequate service delivery, lack of information about immunization and limited number of vaccinators were found to be the key reason for poor performance of immunization and for large number of cases reported each year due to the deficiency of second and third booster dose.

## Introduction

Many epidemics are caused by poliovirus in the last three centuries. About 100,000 new polio cases are reported each year worldwide. Europe and North America were the targets of Epidemic poliomyelitis in 1890s [1]. Now a day most of these cases occur in Asia and Africa [2]. German physician Jakob Heine recognized Poliomyelitis as a distinct condition for the first time in 1840 [3]. Poliovirus was identified by Austrian physicians Karl Landsteiner and E. Popper in 1908 [2]. The three serotypes 1, 2, and 3 infect cells via a specific receptor, PVR (polio virus receptors): CD-155. These receptors are only present in human cell that is why humans are the only reservoirs of this virus [4]. The serological relationship is present between serotype 1 and serotype 2. This is conferred by significant protection against type 2 by the antibodies which were produced against serotype 1 [5]. Immunologically Type 2 is broad and it is the first serotype to disappear during vaccination campaigns. Albert Sabin and Jonas Salk developed effective vaccines against Polio virus in the early 1960s with different approaches. The Sabin oral vaccine which contains live attenuated polio virus is superior to the Salk inactivated vaccine in two ways, firstly it is easily administered; secondly it provides a long-lasting immunization [6]. Pakistan use trivalent OPV that contain all the three serotypes in attenuated form.

Following the successful eradication of small pox, World Health Assembly (WHA) decided to target polio virus eradication [7]. By the efforts of World Health Organization (WHO) program Global Eradication of paralytic poliomyelitis has been eradicated from America, Western Europe, and many other regions of the world [8,9]. Expended program on immunization began in Pakistan in 1976 and expanded countrywide by 1978. Both regionally and worldwide EPI has a significant impact on poliomyelitis eradication performance. Pakistan is achieving immunization targets set globally and has made progress towards achieving Millennium Development Goal 4. But lack of parent knowledge, limited access to immunization services and poor managements are present as hard barriers in front of immunization progress. Every year vaccines for approximately 5.8 million children are procured by the program [10]. More than 30 million children are immunized in every round of polio supplemental immunization activities. EPI is the exclusive provider of immunization in Pakistan and about 3% of immunization is provided by private sector [11]. Each year Over 6000 permanent centers and more than one million outreach and mobile vaccination sessions provides immunization services. Over 10,000 vaccinators and approximately 6000 lady health visitors (LHVs) are assigned in these centers. About 100,000 lady health workers (LHWs) also aid in routine and supplementary immunization activities. The current routine immunization schedule for children is described in Table 1.

**Table 1:** Routine Immunization Schedule for Children EPI Pakistan

Name of vaccine	No. of doses	Age of administration
BCG	1	At birth
Trivalent OPV	4	At birth, 6, 10 and 14 weeks
Measles	2	At 9 month and 2nd year of life
Pentavalent (DPT-Hepatitis B)	3	At 6,10 and 14 weeks
DPT	3	6.10 and 14 Weeks of Birth

## Materials and methods

Several Government documents, survey reports and unpublished program documents were studied and online searches were made to find literature on EPI Pakistan. World Health Organization (WHO), United Nations Children's Fund (UNICEF) and other websites were also explored. The EPI program official database was also analyzed for this study. SPSS 16 and Microsoft Excel 7 were used for the statistical analysis, tabulation and compiling of collected data.

## Results

### 1. Immunization

#### OPV III immunization in all provinces of Pakistan

A "fully immunized child" is one who has received at least 1 dose of Bacilli Calmette-Guérin (BCG) vaccine, 3 doses of oral polio vaccine (OPV), DPT3 and measles1 vaccine. EPI programs target is to immunize children of 0-11 months against seven EPI target diseases. According to EPI surveys 2001, Khyber Pakhtunkhwa was the best performing province with 89% immunization (OPV III) in fighting against polio. The lowest immunization results were in Baluchistan and Gilgit Baldistan with 52% and 34.6% immunization respectively. The EPI surveys are regularly conducted in Pakistan by the Ministry of the Health in collaboration with WHO and other organizations that are active in fight against polio. The main aim of the surveys is to get data about the immunization, to identify the regions that are at the risk so that the special consideration should be given to these areas in planning the programs, in future.

**Figure 1:**
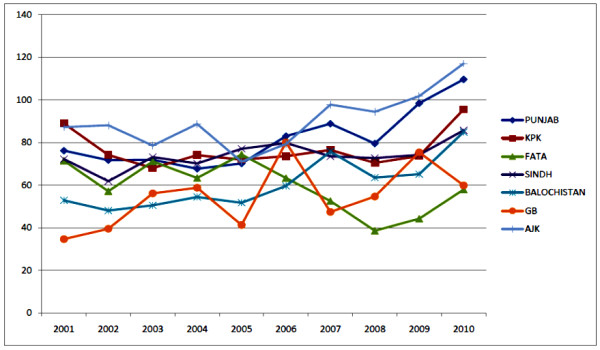
**Percentage immunization of Polio in different provinces of Pakistan in the last decade**.

Figure 1 shows the percentage immunization of OPV III vaccination in different provinces of Pakistan during the last decade (2001-2010). The vaccination campaigns conducted from 2002-2004 shows approximately the same results as in previous exercise in 2001. Azad Jammu and Kashmir (AJK) and KPK were the best performing provinces with 88% and 74% immunization and Baluchistan and Gilgit Baldistan having the lowest immunization 54%, 58% respectively. The main reason for the lowest immunization in Baluchistan and Gilgit Baldistan are the poor infrastructure, low education rate, lack of political well and highly diverse and inaccessible population. In 2005, over all reported immunization was 65%, the immunization campaign highly affected due to severe earthquake in the northern area of the Pakistan, due to which a huge population migrated to the other areas of the country. The surveys conducted during 2006 to 2010 in all the province of Pakistan (Figure 1) shows that vaccine immunization has been greatly improved in door-to-door immunization campaigns.

AJK and Punjab remains the highest performing provinces. As compared to the other parts of the country lowest immunization was observed in FATA due to fight against terrorism and security concerns in that region. As appeared from the data most of the cases are clustered at its borders with Afghanistan. Pakistan shares common epidemiological block with Afghanistan. So both countries should synchronize their efforts for fight against polio.

#### Immunization survey of OPV I, II and III in Pakistan

**Figure 2:**
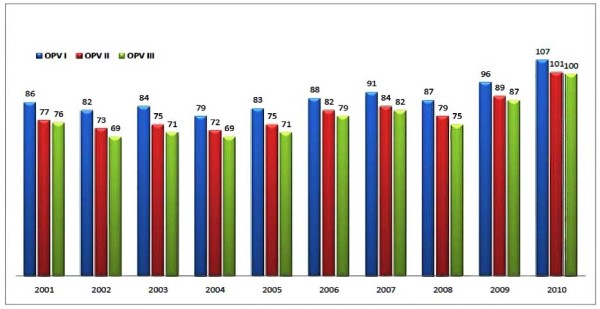
**OPV I, OPV II, OPV III immunization in Pakistan**.

Each year about 10-20% children fail to receive their third dose against poliomyelitis. In 2001, 86% of the target population of 0-11 months and above received their first dose against but only 76% received OPVIII (Figure 2). Similarly 13% children in 2002 and 2003, 10-12% in 2004 and 2005 and up to 7% children in 2010 could not receive their third dose against polio. This could possibly be the reason why polio has not being eradicated from Pakistan. The country has made significant improvement in EPI immunization as compared to its neighboring countries, but there is need to adopt a more aggressive implementation strategy to compete with other countries of the region.

### 2. Surveillance

**Figure 3:**
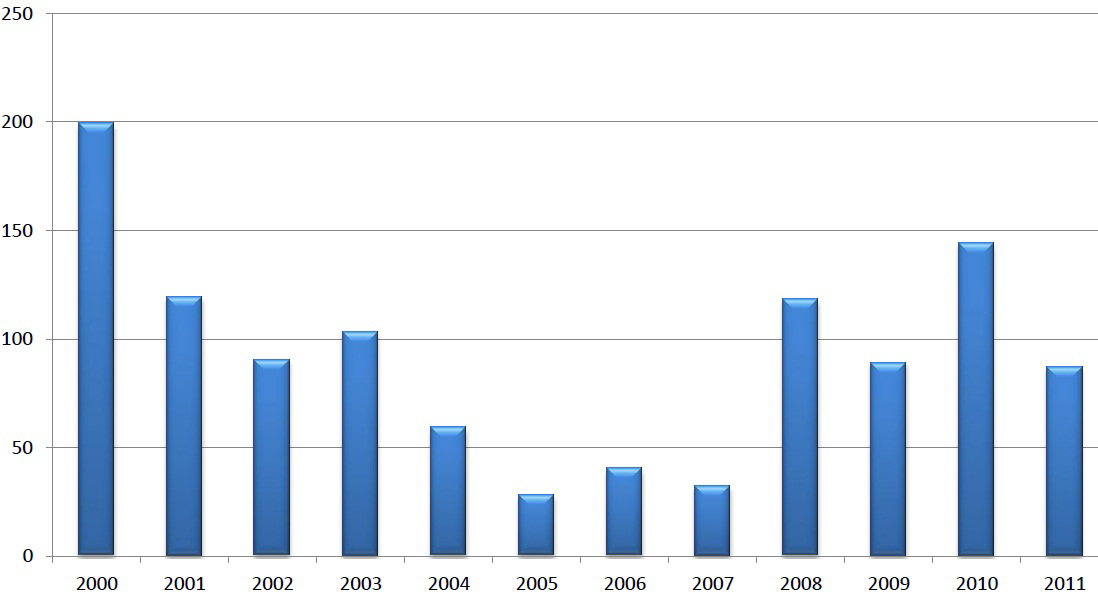
**No of confirmed polio cases in the last decade in Pakistan**.

**Figure 4:**
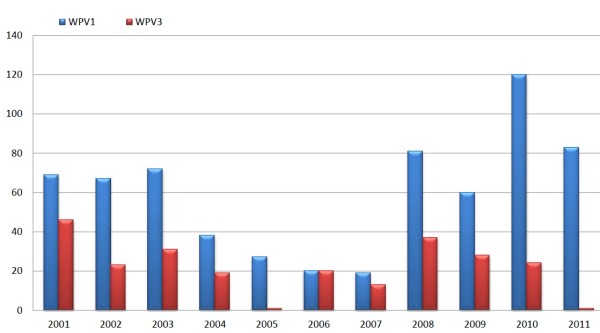
**Year Vise Distribution of Type 1 and type 3 Polio Virus**.

Pakistan held 5 rounds of national immunization days (NID) and two sub NIDs in 2001, but still 119 cases of polio were reported (Figure 3). 20 cases were confirmed in Baluchistan (8 cases in Quetta), 18 in Punjab, 25 cases registered in Sindh, and 22 in KPK (4 cases in the Peshawar District). In 2002 the number of cases decreased from 119 to 90. Most cases were reported from northern Sindh, southwestern Punjab, the Peshawar district and the southern area of Khyber Pakhtunkhwa (KPK) [12]. In general more polio cases were reported in 2003 as compared to 2002, 103 cases were reported in this year. But, in the start of second half of 2003, the number of cases began to reduce, that is 55 cases were reported in second half of 2003 compared to 60 during the same period in 2002. In 2004, a total of 59 polio cases were confirmed, 38 caused due to WPV1 and 19 due to WPV3. During 2003 to 2004, the number of reported polio cases reduced about 50%, with 46 cases due to serotype 1 (WPV1) and seven due to WPV3 (Figure 4) [13]. Punjab, the well populated province of Pakistan, reported 3 polio cases during the first half of 2004 and 14 cases, mostly from southern Punjab, during the second half. The genetic sequencing shows that certain viruses detected in Punjab were directly imported from Sindh and KPK. But analysis of genetic sequencing also suggests that at least two WPV1 and one WPV3 lineage were identified in Punjab during 2004.

Twenty eight polio cases were reported in 2005. Out of these twenty seven cases were WPV1, and one was WPV3 (from Quetta district in the Baluchistan province). In 2006 the number of reported polio cases from 17 districts was increased from 28 to 40. Of the 40 cases, 20 were caused due to WPV1 and 20 due to WPV3. Most of the WPV1 cases were identified from Sindh Province or from security-compromised areas in KPK [14]. In 2007 the number of polio cases reduced from 40 (cases reported in 2006) to 32 cases (reported from 18 districts), 19 cases were caused due to WPV1 and 13 due to WPV3. While this number greatly increased during 2008. In this year 118 cases were reported, out of which 81 cases were caused due to WPV1 and 37 due to WPV3 [15]. This is because of the failure to conduct SIAs in the conflict affected border areas of Pakistan and Afghanistan; large areas of KPK and FATA were too dangerous to conduct SIAs. Also political and management issues in Sindh and Baluchistan badly affected the supervision and conduct of immunization programs. In 2009, Surveys reported 89 polio cases (60 WPV1, 28 WPV3, and one mixed WPV1/WPV3) [16]. 144 cases were reported (120 WPV1 and 24 WPV3) in 2010. 100 cases were reported from conflict-affected areas, including 23 cases from FATA and 73 cases from other parts of KPK [17]. Until now 87 new polio cases are reported this year. 35 cases are reported from Baluchistan, one from Gilgit-Baldistan one from Punjab, 17 from Sindh, 26 from Fata and seven from Khyber-Pakhtunkhwa. Of the 17 cases reported in Sindh; Hyderabad, Umerkot, Tando Allahyar and Jamshoro reported one case each, Badin, Tando Muhammad Khan and Sanghar reported two each, Thatta reported three and Karachi reported four [18]. Case report survey of 2009 shows that all the provinces were at the risk and the possible reason was the heavy flood, which badly affected more than 50% area of the country. The affects of the recent flood possess potential risk on a number of fronts including damaged health infrastructure, increased pressure on management and compromised quality of the drinking water, sanitation and increased displaced population. High risk population groups included migrants, internally displaced persons (IDP) due to war against terrorism and afghan refugees. If we focus our study on the case report of polio month wise, we found that the polio increases in some months (July to November) due to environment effects, use of poor quality drinking water and unhygienic conditions resulted in previous summer. The concerned authority should have to design a plan with the top political commitment to prevent Pakistan from becoming the last remaining reservoir of polio virus circulation on the globe.

## Discussion

Pakistan's current immunization strategies against poliomyelitis include routine administration of oral poliovirus vaccine (established in 1979) at birth and at ages 6, 10, and 14 weeks and on annual national immunization days(NIDs) (began in 1994) during which 2 doses of OPV are administered at 4- to 6-week intervals for all 5 years old children.

All surveys conducted from 1995 to 2007 for immunization in Pakistan showed that only about half of the targeted children were fully immunized in spite of the high immunization claimed in the administrative reports [11,19-22]. The main cause for the weak performance of EPI in Pakistan was the limited access to the immunization services [11,22-26]. The failure to reach all children, especially in high risk areas, with sufficient doses of vaccine is leading to continued transmission of poliovirus in Pakistan. Pakistan is now reporting more cases than the combined total cases of the other three endemic countries: Nigeria, India, and Afghanistan. Children in high risk areas are not receiving adequate number of doses of oral poliovirus vaccine (OPV) due to difficulties in access in areas affected by security problems particularly in FATA and parts of Khyber Pakhtunkhwa. Recent floods have posed an additional challenge in terms of mass displacement of populations and damaged health infrastructure especially in the northern Sindh and southern Punjab [27].

Insufficient number of vaccinators was another main reason for the limited access to service [23,25]. The unequal distribution and limited number of vaccinators has left a great number of the UCs uncovered. EPI-trained LHWs for providing vaccination services can overcome this problem. LHWs have substantial potential for enhancing EPI immunization in their local areas because they are embedded in, and accepted by the community. Lack of awareness about immunization was another main reason for not utilizing the available immunization services [22,28]. Earlier studies showed that lack of information among parents about the benefits of vaccination was one of the key reasons for the failure to vaccinate their children. Recent study indicates good results in this area [29]. Approximately 84% of mothers agreed that vaccination helps to keep their child healthier. Approximately 63.3% mothers come across problems when their kid misses 1 or 2 doses and one-third were in favor of "No problem" if vaccine is missed [23].

## Conclusion

In spite of numerous achievements, such as the addition of new vaccines and raising immunization to over 100% in some areas, EPI is still struggling to reach its polio eradication goals. Inadequate service delivery, Lack of information about immunization and inadequate number of vaccinators were found to be one of the main reasons for this poor performance and large number of cases reported each year. Some specific efforts can be taken to improve EPI services in Pakistan. Providing regular EPI services through all existing health facilities, establishing an EPI centre in all UCs, reorganizing the available vaccinators and engaging all the skilled manpower including LHWs for vaccination, could raise community access and compliance to the service and thus raises immunization significantly.

## Competing interests

The authors declare that they have no competing interests.

## Authors' contributions

MS and AS conceive the idea of this work. MKK, ZS and MBS helped SS and FM in literature review and data collection. MS and SS perform statistical analysis. MS and FM critically reviewed and finalized the manuscript. AS provided all facilitates to complete this work. All authors read and approved the final manuscript.
